# Factor VII Deficiency and Second Trimester Abortion: A Case Report

**DOI:** 10.7759/cureus.37039

**Published:** 2023-04-02

**Authors:** Katie P Nguyen, Tamara Lynne B Aqui, Honey Milestone

**Affiliations:** 1 Family Medicine, Riverside Community Hospital, Riverside, USA; 2 Internal Medicine, Riverside Community Hospital, Riverside, USA; 3 School of Medicine, University of California, Riverside, USA

**Keywords:** surgery, hematology, trauma, induced abortion, factor vii deficiency

## Abstract

The prevalence of factor VII deficiency (F7D) is 1 in 500,000. Due to its rarity, the management of bleeding disorders in pregnancy is not well established. We examine a case of an 18-year-old (gravida 1, para 0) woman at approximately 19 weeks gestation with a known history of F7D who presents after a motor vehicle accident. Fetal demise was confirmed necessitating a medical induction. She also had multiple fractures requiring surgical intervention. A multidisciplinary team consisting of orthopedic surgery, obstetrics and gynecology, and hematology/oncology was consulted for optimal timing of factor VII replacement prior to procedures. The patient underwent successful left tibial intramedullary nailing with minimal bleeding. She received factor VII and tolerated an uncomplicated vaginal delivery. Her postpartum and postoperative courses were uncomplicated, requiring one unit of packed red blood cells. The patient was discharged on postpartum day three. Management of this second-trimester abortion with a history of F7D was possible with effective communication and the organization of a multidisciplinary team to account for the risk of thrombosis versus hemorrhage and the availability of factor VII replacement therapy.

## Introduction

Factor VII deficiency (F7D) is an autosomal recessive rare inherited coagulation disorder (RICD) affecting the extrinsic pathway of the coagulation cascade. The prevalence of F7D is 1 in 500,000 and 1 in 2 million for homozygous forms. Due to the rarity of RICDs, the type and severity of bleeding symptoms, underlying molecular defects, and management of bleeding disorders are not well established [1]. Balancing the risk of thrombosis and hemorrhage, especially in emergent situations, remains a challenge in managing pregnancies complicated by F7D as its clinical presentation correlates poorly with factor VII levels and the role of factor replacement therapy is not clearly defined [2]. However, heterozygotes can still have mild bleeding symptoms and are at increased bleeding risk during surgery, invasive procedures, and pregnancy [3]. This case reports on a young patient who was heterozygous for F7D at approximately 19 weeks gestation and who presents after a motor vehicle accident with a second-trimester abortion.

## Case presentation

The patient is an 18-year-old (gravida 1, para 0) woman at 19 weeks and 4 days gestation who presented to the emergency department after a motor vehicle accident. As the restrained driver, traveling at approximately 13.4 m/s, another vehicle going at approximately 22.4 m/s collided with the patient’s passenger side of the vehicle. Airbags were deployed, and the patient was extricated from the vehicle. The patient’s Glasgow Coma Scale was 15, and on physical exam, she was normotensive, without signs of hemorrhage, and oxygen saturation was in the high 90s on room air. Primary and secondary surveys were completed, and no immediate intervention was required. Bedside Focused Assessment with Sonography for Trauma was negative, except for weak fetal cardiac activity. A formal obstetric ultrasound was ordered confirming placental abruption and fetal demise. Imaging was significant for comminuted displaced fracture of the left mid tibia and fibula, left clavicle fracture, and a small left pneumothorax.

The patient was diagnosed with F7D at the age of two after an episode of epistaxis. She reported a history of heavy menstrual bleeding but otherwise denied other sources of spontaneous bleeding. The patient had previously undergone an appendectomy and tonsillectomy without hemostatic complications. According to her hematologist, she was heterozygous F7D, and her last known levels were 40%. Other family, maternal, and obstetric histories were unremarkable.

The management of this patient’s care involved the collaboration of various specialty teams including emergency medicine, trauma surgery, obstetrics and gynecology, orthopedic surgery, critical care, hematology/oncology, pharmacy, physical therapy, occupational therapy, and rehabilitation services. A multidisciplinary team meeting was held to assess the timing of orthopedic surgery versus medical induction of labor and the risk of thrombosis versus hemorrhage. The patient underwent left tibial intramedullary (IM) nailing with orthopedic surgery on hospital day two (HD#2) and tolerated the procedure well with minimal bleeding. The patient did not require operative management of her left clavicle fracture. Hematology/oncology was consulted in preparation for medical induction of labor who recommended the administration of one dose of coagulation factor VIIa at 40-50 mcg/kg (approximately 2 mg) every 12 hours to 24 hours with frequent monitoring prior to the procedure. They also recommended against the use of low molecular weight heparin (LMWH) prior to delivery as it counteracts the hemostasis efforts. The patient was at increased risk of clotting, stroke, deep vein thrombosis, and pulmonary embolus (PE) with factor VII replacement but also paradoxically at high risk for postpartum hemorrhage from F7D. She received misoprostol 800 mcg followed by 400 mcg three hours later which successfully led to an uncomplicated medical abortion on HD#3 in the ICU. There was no evidence of fetal movement, breathing, or pulse, and fetal demise was confirmed. The estimated blood loss was 200 ml. Placental pathology showed a 4-cm retroplacental hematoma and separate fragments of clotted blood, consistent with placental abruption. Her postpartum course was uncomplicated; thus, the decision was made to start the patient on LMWH after she received coagulation factor VIIa. The pneumothorax resolved spontaneously. Her hemoglobin was 6.6 gm/dL on HD#4 after the medical abortion which was a drop from 11.0 gm/dL on the initial presentation. She received one unit of packed red blood cells (pRBC), and her hemoglobin improved to 9.4 gm/dL on HD#5. Four doses of coagulation factor VIIa 2 mg were given to the patient in total. The patient was monitored and remained stable for subsequent discharge on HD#5, postpartum day three (PPD#3). Table 1 summarizes the key events and labs given on each day of hospitalization.

**Table 1:** Timeline of patient’s hospitalization and key events The PT is expected to be prolonged. This patient’s PT was only mildly elevated and actually decreased after receiving coagulation factor VIIa. This patient’s platelet levels were within normal limits on admission, which fluctuated throughout the hospitalization. HD: hospital day, PPD: postpartum day, Hgb: hemoglobin, Plt: platelet, PT: prothrombin time, IM: intramedullary, pRBC: packed red blood cells, LMWH: low molecular weight heparin.

Day	HD#1 (admission)	HD#2	HD#3/PPD#1	HD#4/PPD#2	HD#5/PPD#3 (discharge)
Hemoglobin	11.0	7.8	7.1	6.6	9.4
Platelets	210	121	120	108	134
Prothrombin time	13.7	14.7	Not done	8.5	Not done
Surgical procedures	None	IM nailing with orthopedic surgery	Medical abortion	None	None
Medications given	None	Coagulation factor VIIa 2 mg, misoprostol 800 mcg and 400 mcg	LMWH	1 unit pRBC, LMWH, coagulation factor VIIa 2 mg	LMWH, coagulation factor VIIa 2 mg x2

## Discussion

Given the emergent nature of this patient’s presentation with a history of F7D, one requires an understanding of the disorder with a systematic approach through a thorough history and physical examination in collaboration with various specialty services to prevent hematologic emergencies. This patient is a heterozygous carrier of F7D resulting in a quantitative deficiency where plasma levels of the factor are reduced. Generally, heterozygous gene mutations have factor VII levels between 20% and 60%, whereas homozygous levels tend to be less than 10%, but these levels can vary based on the specific mutation [2]. Heterozygotes are usually asymptomatic carriers that can still have mild bleeding with increased bleeding risk during procedures and pregnancy; however, homozygotes and compound heterozygotes are more likely to manifest the disease condition. F7D affects both males and females, but females are at increased risk of bleeding due to menstruation and childbirth. The age of bleeding onset can vary greatly. According to the rare bleeding disorders registry, the median age at diagnosis was seven years old with a range from birth to 73 years old [4]. The most common presentations were excessive bleeding of the skin, mucus membranes (epistaxis), muscles, or joints, after trauma or invasive procedures (e.g., circumcisions, tooth extractions), menstruation, childbirth, and in postpartum [5]. This patient was diagnosed with F7D when she was two years old after an episode of epistaxis and had regular follow-up visits with her outpatient hematologist.

In other bleeding disorders, heterozygotes usually do not have severe bleeding because the normal allele of the factor gene contributes 50% of the normal levels which is sufficient for hemostasis. However, the clinical severity of F7D correlates poorly with factor VII levels which can range from 50% to 200%, and the role of factor replacement therapy is not clearly defined. The half-life of factor VII is 4-6 hours with a targeted level of 15-20% required at minimum to prevent spontaneous bleeding [1]. In a study of 28 patients with severe F7D with levels at 2% or less, the most common symptoms were epistaxis and heavy menstruation, but hemarthrosis and muscle hematoma were less frequent. About 55% of patients who underwent surgery without factor VII replacement had postoperative bleeding that required blood transfusions [6]. Another study followed three African-American females with severe factor VII levels at less than 1% to 9% who did not have excessive bleeding after various surgical procedures which supported that clinical history is the better predictor of bleeding tendencies. Heterozygotes with levels above 10% are unlikely to have severe bleeding episodes [7]. However, a review of 717 patients in the Greifswald Registry of F7D showed that 93 of 499 (19%) had spontaneous bleeding with a factor VII level of 39% (hemarthrosis 4%, epistaxis 54%, gum bleeding 14%, easy bruising 38%, hematoma 23%, hematuria 5%, and heavy menstruation 42%) [5]. This patient’s last known factor VII level was 40%, and she tolerated IM nailing with orthopedic surgery well. Her clinical history was pertinent for a prior appendectomy and tonsillectomy without hemostatic complications.

According to a systematic review of the literature on the management of pregnancies complicated by F7D, the hemorrhage rates are equivalent in patients with and without hemostatic prophylaxis. The recommendation is to consider prophylaxis use of recombinant factor VII or factor VII concentrate as part of an individualized discussion in the context of prior bleeding complications and mode of delivery (e.g., cesarean sections). To improve patient outcomes, hemostatic agents should be available in case of hemorrhage or surgical intervention [2]. Possible sources of bleeding to anticipate include the placental implantation site, episiotomy site, lacerations to the birth canal, and surgical trauma with cesarean delivery [8]. Since of the short half-life of recombinant factor VII, continuous infusion therapy may be an option for surgical candidates [9]. It would be beneficial to know the patient’s starting factor VII level prior to replacement therapy. The hemostatic prophylaxis agent of choice is recombinant activated factor VII. Because it is not derived from human plasma, the risk of blood-borne pathogens transmissions, such as hepatitis or human immunodeficiency virus, is eliminated. The primary adverse effect of using recombinant factor VII is an increased risk of thrombosis. It is suggested that thromboelastography, which measures whole-blood coagulation, can be used to monitor the efficacy of recombinant factor VII in the prevention of thrombosis [10]. Other traditional considerations include four-factor prothrombin complex concentrates, fresh frozen plasma (FFP), and/or tranexamic acid (TXA). The patient had a combination of pRBCs, FFPs, and TXA available in the event of a massive hemorrhage. She received factor VII replacement and only required one unit of pRBC transfusion after her vaginal delivery. Paradoxically, patients can have thrombosis reported in 3-4% of F7D even those with severe deficiency with or without factor replacement [11]. Pregnancy is associated with 20-1000% increases in factors VII, VIII, IX, X, and XII [12]. This poses a challenge to the utility of thrombosis prophylaxis with or without replacement therapy. The risk of developing thromboembolism is fourfold to fivefold for pregnant or postpartum patients compared to nonpregnant women [13,14]. The decision was made to start this patient on LMWH only after she received coagulation factor VIIa and postpartum.

As mentioned prior, pregnancy itself is a prothrombotic state due to the numerous metabolic, immunological, and hemostatic changes that take place in the body. In pregnancy, plasma volume can expand up to 40%, with RBC expanding only 25%, and platelets also experience a relative decrease due to dilution and sequestration by the placenta. These effects are offset by an increase in coagulation factors. However, in some coagulation disorders, the deficiency of certain factors can still place pregnant patients at risk, especially during the postpartum period. Von Willebrand disease (VWD) is one of the most common bleeding disorders found in the general population and affects upwards of 50,000 deliveries annually. Patients with type 1 VWD disease attain normal factor levels as pregnancy progresses, while those with type 2 and 3 VWD disease are more at risk for hemorrhage, especially during the first 24 hours postpartum. Patients with hemophilia A and B (factor VIII and factor IX deficiencies, respectively) are usually asymptomatic carriers; however, a large proportion still experiences coagulation factor levels far below normal during pregnancy. While factor VIII levels increase throughout the pregnancy gestation, factor IX levels do not increase as much, which makes supplementation to normal non-pregnancy levels a necessity if the patient has a history of bleeding or low factor levels [15]. Management of pregnant patients with RICDs remains a challenge to balance the risk of thrombosis and hemorrhage. Hematologic emergencies contribute to morbidity and mortality in the pregnant patient population. In order to improve patient outcomes, one should be aware, anticipate, and recognize potentially hazardous events and obstetric emergencies. The types of coagulopathies in pregnancy include prothrombotic or microangiopathic events (hemolysis elevated liver enzymes low platelets or HELLP syndrome, preeclampsia, eclampsia), hemolytic uremic syndrome, thrombotic thrombocytopenia purpura, disseminated intravascular coagulopathy (DIC), deep venous thrombosis, pulmonary embolism, and recurrent pregnancy losses. Bleeding events including antepartum hemorrhage and postpartum hemorrhage are always on the differential as well [16]. A thorough history of past bleeding episodes especially in childhood, family history of bleeding, menarche and menstruation, pregnancy, and prior hematologic workup is crucial. Running laboratory tests including coagulation studies, complete blood count, and current factor VII level would guide management. The challenge is obtaining the necessary and pertinent history and laboratory values in a timely manner, especially in emergent cases. This patient was able to tell the medical team that she has F7D, but her outpatient hematologist and the inpatient hematologist/oncologist both played a crucial role in guiding the management. A prolonged prothrombin time (PT) suggests F7D or underlying liver disease affecting the extrinsic pathway of coagulation. A prolonged partial thromboplastin time (PTT) suggests a deficiency of the intrinsic pathway instead. If both values are prolonged, there may be a defect in the global coagulation system such as DIC or other sources of hemorrhage. In contrast, a shortened PT or PTT could indicate a prothrombotic state such as preeclampsia or early DIC [16]. It is not uncommon for pregnant patients to develop thrombocytopenia toward the end of the pregnancy, and thus pregnancy itself is thought to be a possible trigger for thrombotic thrombocytopenic purpura [17]. Monitoring the patient’s clinical status, vital signs, and pertinent laboratory values narrow down the differential diagnosis. This patient’s PT was only mildly elevated and actually decreased after receiving coagulation factor VIIa. This patient’s platelet levels were 210 mcL on admission which were normal and fluctuated throughout the hospitalization. Studies show that heterozygous F7D patients can have a significant increase in factor VII levels throughout pregnancy. However, early pregnancy losses are more likely to have excessive hemorrhage when compared to term deliveries because of the insufficient rise in the factor VII level. Having appropriate follow-up and close monitoring of factor VII levels throughout pregnancy would improve outcomes [18].

## Conclusions

The management of pregnant patients with F7D remains a challenge to clinicians in balancing the risk of thrombosis and hemorrhage. The level of factor VII does not always correlate with clinical severity and there are no concrete guidelines for the indications or dosing criteria for factor VII replacement therapy. Taken together, management of the delivery for pregnant patients with F7D should be addressed on a case-by-case basis at centers where a multidisciplinary team with expertise is readily available. Managing this patient’s bleeding risk depends on bleeding severity, clinical history, the initial level of factor VII, and the availability of factor replacement products, ideally recombinant activated factor VII. Patients wishing for further pregnancies should continue to require regular visits with outpatient hematology to monitor factor VII levels, as having F7D could increase the chances of having recurrent miscarriages. Regular follow-up with therapy and counseling services, including mental health and reproductive health, and OBGYN would also benefit and empower the patient for future fertility plans.
